# Risk Factors, Diagnosis and Management of Chyle Leak Following Esophagectomy for Cancers: An International Consensus Statement

**DOI:** 10.1097/AS9.0000000000000192

**Published:** 2022-08-29

**Authors:** Sivesh K. Kamarajah, Manjunath Siddaiah-Subramanya, Alessandro Parente, Richard P. T. Evans, Ademola Adeyeye, Alan Ainsworth, Alberto M. L. Takahashi, Alex Charalabopoulos, Andrew Chang, Atila Eroglue, Bas Wijnhoven, Claire Donohoe, Daniela Molena, Eider Talavera-Urquijo, Flavio Roberto Takeda, Gail Darling, German Rosero, Guillaume Piessen, Hans Mahendran, Hsu Po Kuei, Ines Gockel, Ionut Negoi, Jacopo Weindelmayer, Jari Rasanen, Kebebe Bekele, Guowei Kim, Lieven Depypere, Lorenzo Ferri, Magnus Nilsson, Frederik Klevebro, B. Mark Smithers, Mark I. van Berge Henegouwen, Peter Grimminger, Paul M. Schneider, C. S. Pramesh, Raza Sayyed, Richard Babor, Shinji Mine, Simon Law, Suzanne Gisbertz, Tim Bright, Xavier Benoit D’Journo, Donald Low, Pritam Singh, Ewen A Griffiths

**Affiliations:** From the * Department of Upper Gastrointestinal Surgery, Queen Elizabeth Hospital Birmingham, University Hospitals Birmingham NHS Trust, Birmingham, United Kingdom; † Institute of Cancer and Genomic Sciences, College of Medical and Dental Sciences, University of Birmingham, Birmingham, United Kingdom; ‡ Department of Surgical Education, University of Melbourne, Melbourne, VIC, Australia; § Department of Hepatobiliary Surgery, Queen Elizabeth Hospital Birmingham, University Hospitals Birmingham NHS Trust, Birmingham, United Kingdom; ∥ The Division of Surgical Oncology, Department of Surgery, Afe Babalola University (ABUAD), Nigeria; ¶ Odense University Hospital, Denmark; # Department of Gastrointestinal Tumors, National Cancer Institute, Mexico City, MX; ** Laikon General Hospital, Greece; †† Section of Thoracic Surgery, Department of Surgery, University of Michigan, Ann Arbor, MI; ‡‡ Department of Thoracic Surgery, Medical Faculty, Ataturk University, Erzurum, Turkey; §§ Department of Surgery, Erasmus University Medical Center, Rotterdam, the Netherlands; ∥∥ Department of Surgery, Trinity College Dublin, St James’s Hospital, Dublin, Ireland; ¶¶ Thoracic Service, Department of Surgery, Memorial Sloan Kettering Cancer Center, New York, NY; ## Department of Surgery, San Raffaele Hospital, Vita-Salute San Raffaele University, Milan, Italy; *** Gastroenterology Department, University of São Paulo, São Paulo, Brazil; ††† Department of Thoracic Surgery, Toronto General Hospital, Toronto, ON, Canada; ‡‡‡ Hospital San Ignacio-Universidad Javeriana, Colombia; §§§ CNRS, Inserm, CHU Lille, UMR9020-U1277—CANTHER—Cancer Heterogeneity Plasticity and Resistance to Therapies, University of Lille, Lille, France; ∥∥∥ Hospital Sultanah Aminah, Johor Bahru, Malaysia; ¶¶¶ Taipei Veterans General Hospital, Taiwan; ### Department of Visceral, Transplant, Thoracic and Vascular Surgery, University of Leipzig, Germany; **** Emergency Hospital of Bucharest, Romania; †††† General and Upper G.I. Surgery Division, University of Verona, Verona, Italy; ‡‡‡‡ Department of Thoracic Surgery, Heart and Lung Center, Helsinki University Hospital, University of Helsinki, Helsinki, Finland; §§§§ School of Medicine, Department of Surgery, Madda Walabu University Goba Referral Hospital, Bale-Goba, Ethiopia; ∥∥∥∥ National University Hospital, University Surgical Cluster, Singapore; ¶¶¶¶ Department of Thoracic Surgery, University Hospitals Leuven, Leuven, Belgium; #### Department of Thoracic Surgery and Upper Gastrointestinal Surgery, McGill University, Montreal, Canada; ***** Karolinska Institutet and Karolinska University Hospital, Stockholm, Sweden; ††††† Department of Surgery, Princess Alexandra Hospital, University of Queensland, Brisbane, QLD, Australia; ‡‡‡‡‡ Department of Surgery, Amsterdam UMC, University of Amsterdam, Cancer Center Amsterdam, Amsterdam, the Netherlands; §§§§§ Department of General-, Visceral- and Transplant Surgery, University Medical Center of the Johannes Gutenberg University, Mainz, Germany; ∥∥∥∥∥ Department of Digestive and Oncological Surgery, Hirslanden Medical Center, Zurich, Switzerland; ¶¶¶¶¶ Department of Surgical Oncology, Tata Memorial Centre, Homi Bhabha National Institute, Mumbai, India; ##### Department of Surgical Oncology, Patel Hospital, Karachi, Pakistan; ****** Department of General Surgery, Middlemore Hospital, Auckland, New Zealand; †††††† Department of Gastroenterological Surgery, Cancer Institute Hospital of Japanese Foundation for Cancer Research, Tokyo, Japan; ‡‡‡‡‡‡ Department of Surgery, The University of Hong Kong, Queen Mary Hospital, Hong Kong SAR, China; §§§§§§ Department of Oesophago-gastric Surgery, Flinders Medical Centre, Australia; ∥∥∥∥∥∥ Department of Thoracic Surgery, Aix-Marseille University, North Hospital, Marseille, France; ¶¶¶¶¶¶ Department of Thoracic Surgery, Virginia Mason Medical Center, Seattle, Washington, DC; ###### Regional Oesophago-Gastric Unit, Royal Surrey County Hospital NHS Foundation Trust, Guildford, United Kingdom.

**Keywords:** esophagectomy, chyle leaks, outcomes

## Abstract

This Delphi exercise aimed to gather consensus surrounding risk factors, diagnosis, and management of chyle leaks after esophagectomy and to develop recommendations for clinical practice.

**Background:**

Chyle leaks following esophagectomy for malignancy are uncommon. Although they are associated with increased morbidity and mortality, diagnosis and management of these patients remain controversial and a challenge globally.

**Methods:**

This was a modified Delphi exercise was delivered to clinicians across the oesophagogastric anastomosis collaborative. A 5-staged iterative process was used to gather consensus on clinical practice, including a scoping systematic review (stage 1), 2 rounds of anonymous electronic voting (stages 2 and 3), data-based analysis (stage 4), and guideline and consensus development (stage 5). Stratified analyses were performed by surgeon specialty and surgeon volume.

**Results:**

In stage 1, the steering committee proposed areas of uncertainty across 5 domains: risk factors, intraoperative techniques, and postoperative management (ie, diagnosis, severity, and treatment). In stages 2 and 3, 275 and 250 respondents respectively participated in online voting. Consensus was achieved on intraoperative thoracic duct ligation, postoperative diagnosis by milky chest drain output and biochemical testing with triglycerides and chylomicrons, assessing severity with volume of chest drain over 24 hours and a step-up approach in the management of chyle leaks. Stratified analyses demonstrated consistent results. In stage 4, data from the Oesophagogastric Anastomosis Audit demonstrated that chyle leaks occurred in 5.4% (122/2247). Increasing chyle leak grades were associated with higher rates of pulmonary complications, return to theater, prolonged length of stay, and 90-day mortality. In stage 5, 41 surgeons developed a set of recommendations in the intraoperative techniques, diagnosis, and management of chyle leaks.

**Conclusions:**

Several areas of consensus were reached surrounding diagnosis and management of chyle leaks following esophagectomy for malignancy. Guidance in clinical practice through adaptation of recommendations from this consensus may help in the prevention of, timely diagnosis, and management of chyle leaks.

Chyle leaks following esophagectomy for cancer have a reported incidence ranging from 1% to 9%.^1,2^ Mediastinal dissection with damage to the duct or branches, or failure of ligation are responsible for a chylothorax.^3,4^ There is wide variation in the anatomic location and patterns of drainage of the abdominothoracic lymphatic systems. In the abdomen,^5,6^ iatrogenic damage to the cisterna chyli during abdominal dissection can lead to chylous ascites as well as chylothorax.

The clinical impact of a chyle leak is substantial with associated morbidity ranging between 0% and 50%^2^ and mortality as high as 10%.^2,7,8^ Furthermore, they are associated with increased hospital costs, and potentially reduced long-term survival.^2^ A reason for the high morbidity and mortality includes the continuous loss of chyle, which is naturally rich in fats, fat-soluble vitamins, enzymes, proteins, and lymphocytes. This may lead to decrease in serum albumin and a significant reduction in peripheral lymphocytes that can in turn, result in malnutrition and immunosuppression. Moreover, loss of pleural fluid may lead to hypovolemia, respiratory failure, sepsis, and malnutrition.^9^

Several reviews have been published on chyle leaks, but none have provided clear recommendations for management in clinical practice.^10^ An international modified Delphi consensus methodology was undertaken among an international group of esophageal surgeons, aiming to identify preoperative risk factors, intraoperative techniques that may be relevant to thoracic duct injury, methods of postoperative diagnosis and management of chyle leaks after esophagectomy.

## METHODS

### Delphi Exercise

**FIGURE 1.**
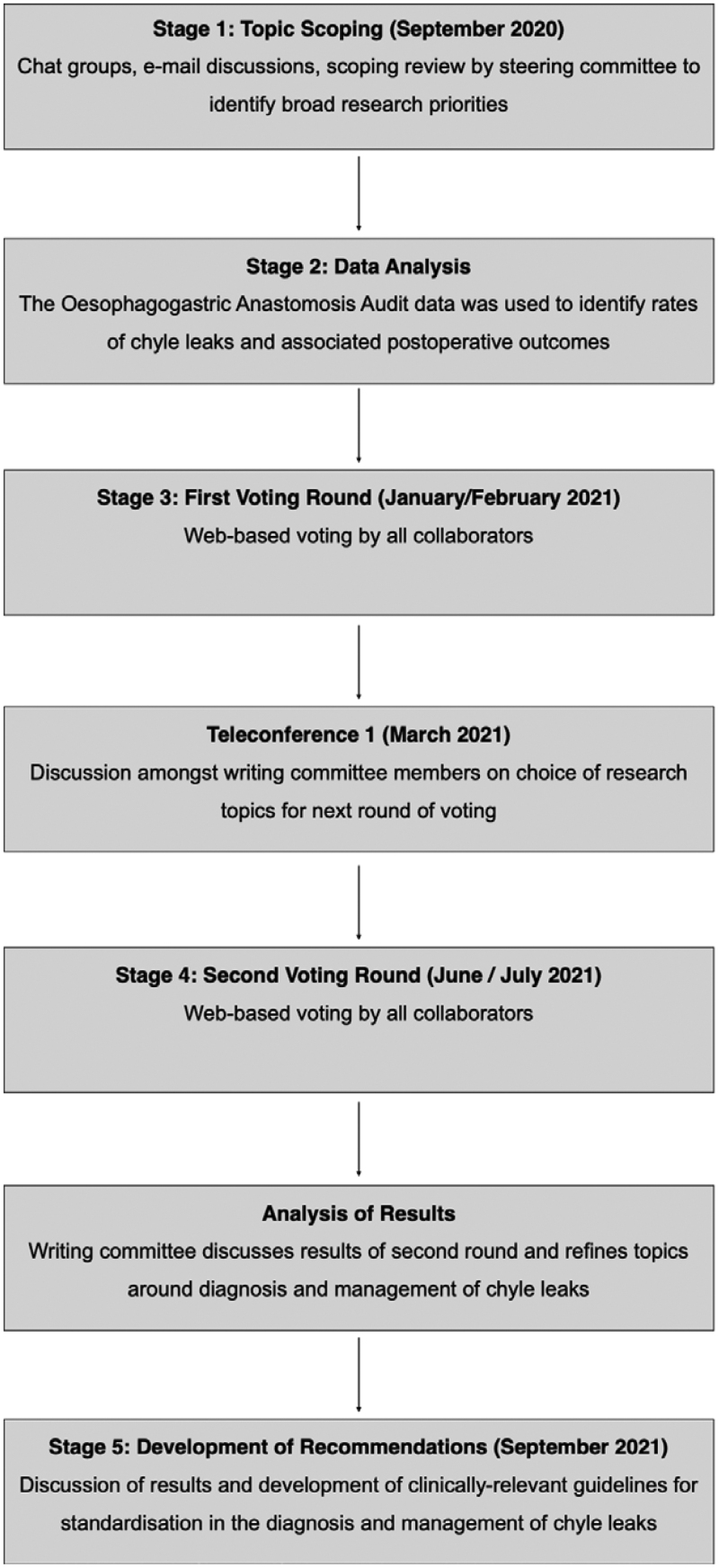
Overview of Delphi exercise to gather consensus surrounding risk factors, diagnosis, and management of chyle leaks after esophagectomy.

The modified Delphi methodology has been described previously.^11,12^ A 5-stage consensus process was designed for this Delphi exercise, including 2 rounds of voting (Figure 1).

#### Stage 1: Evidence-based Mapping

Evidence-based mapping was performed by 3 authors on the December 31, 2020, using PubMed, Embase and the Cochrane Library to identify relevant studies surrounding risk factors, diagnosis, and management of chyle leaks following esophagectomy for cancer. The search (mesh) terms used were “chyle leaks” or “chyle leakage” or “leaks” and, “oesophagectomy,” “esophagectomy,” or “oesophagogastrectomy” and “risk factors,” or “diagnosis,” or “management” individually, or in combination. Literature search strategy is presented in Table S1 (http://links.lww.com/AOSO/A138). Inclusion criteria were as follows: (i) cohort studies or randomized controlled trials reporting risk factors, diagnosis, and management for chyle leaks in human subjects undergoing esophagectomy for cancer; (ii) systematic reviews and meta-analyses; and (iii) articles published in the English language. After excluding duplicates, 2 researchers (SKK, AP) independently reviewed the full texts of identified studies. Reference lists of all included studies were hand searched to identify other potentially relevant studies. Identified topics were then grouped into 5 broad research domains: (i) risk factors for chyle leak; (ii) intraoperative techniques to prevent chyle leaks; (iii) management of chyle leaks, broadly divided into diagnosis, classification of severity, and treatment.

#### Stage 2: Characterizing Impact of Chyle Leak on Outcomes

The Oesophago-Gastric Anastomosis Audit (OGAA) is an international, multicenter prospective cohort study including 141 centers across 41 countries, including patients undergoing esophagectomy for cancer from April 2018 to December 2018 with 90-day follow-up.^13^ The methodology of the study has been previously described.^14^ The main explanatory variable for occurrence of chyle leaks stratified by grade of chyle leaks, as defined according to the Esophageal Complications Consensus Group (ECCG),^15^ is type I (requiring enteral dietary modification), type II (requiring total parenteral nutrition [TPN]), and type III (requiring interventional or surgical treatment). Further division of each grade is possible based on output volume (ie, type A with <1 L daily output and type B with >1 L daily output).^16^ The associated impact of grade of chyle leaks by postoperative outcomes (ie, complications, return to theaters, length of stay, 90-day mortality) were explored.

#### Stages 3 and 4: First and Second Voting Round

A survey consisting of 41 questions were developed by a steering committee (SKK, MS, RPTE, EAG) according to findings from the systematic review and distributed to surgeons from the OGAA collaborative and advertised through specialty organizations’ social media accounts (such as Association of Upper Gastrointestinal Surgeons of Great Britain and Ireland, European Society for Diseases of the Esophagus, and International Society for Diseases of the Esophagus, Australian and Aotearoa New Zealand Gastric and Oesophageal Surgery Association, Society of Gastrointestinal and Endoscopic Surgeons, American Foregut Society). Only consultant or attending surgeons who perform esophageal resections were eligible to complete this survey and responses from trainees were excluded.

For each question, respondents ranked their answer across the 5 research domains (ie, (i) risk factors for chyle leaks, (ii) intraoperative techniques to prevent chyle leaks, (iii) diagnosis (iv), classification of severity, and (v) treatment) using a Likert scale from 1 to 5 (1, strongly disagree; 3, neutral; 5, strongly agree). A free-text comment box was also available at the end of each statement, and an additional section in round 1 of the Delphi questionnaire was included for participants to provide further suggestions. Two complete rounds were conducted for this Delphi exercise with the same participants in both rounds. Results from the first round were analyzed, and any suggestions across the 5 research domains expressed in the free-text section in round 1 were considered for inclusion in the second round. Only complete questionnaires were used in the final analysis and duplicate responses from the same respondent were excluded. Following the 2 rounds of voting, the questions across the 5 research domains were quantified by the proportion of agreement (ie, respondent selecting agree or strongly agree) ≥80%.^17^ Further, thematic analyses of free-text responses for each domain were analyzed and reported.

#### Stage 5: Guidelines Development

Data from the scoping review, voting rounds, and cohort study were used to develop guidelines and consensus with a panel of expert esophageal surgeons. These surgeons were selected by purposeful sampling; working in a high-volume center (ie, ≥60 resections per year) and substantial scientific work on esophageal surgery.^18^ Two focus groups were held with the expert panel, consisting of about 20 surgeons in each to promote discussion in an online setting.^19^ The focus group outline was designed to cover the 5 broad research domains mentioned earlier. The focus groups were moderated by the steering committee and the meeting was rehearsed to ensure standardization. The focus groups were organized in November 2021 using videoconferencing and lasted about 90 minutes each.

### Survey Administration

The survey was administered online using the Research Electronic Data Capture (REDCap) system hosted by the Birmingham Surgical Trials Consortium at the University of Birmingham. Participation in the study was voluntary, with no financial or other remuneration offered. Two reminder e-mails were sent to participants at 2-week intervals after each round of voting. All results and feedback were anonymized, so that no individual or institution could be identified.

### Statistical Analysis

We used Cronbach’s α to evaluate consensus quantitatively among the international expert panel; a Cronbach’s α value of at least 0.80 was representative of an acceptable measure of internal reliability.^20–23^ Categorical variables were compared using the χ^2^ test. Non-normally distributed data were analyzed using the Mann-Whitney *U* test for comparisons across 2 groups, and the Kruskal-Wallis test for comparisons of more than 2 groups. Stratified analyses were performed for responses from the second voting round by: annual department volume (≤50, 51–100, ≥101 procedures) and annual surgeon volume (≤20, 21–50, ≥51 procedures). A *P* value of <0.05 was considered statistically significant and no adjustments were made for multiple comparisons. Heat maps were developed to display the level of consensus (ie, green: ≥80% agreement, yellow: 70%–80%, and red: <70% agreement) across the different research questions.^24^ Data analysis was performed using R version 3.2.2, with TableOne, ggplot2, Hmisc, Matchit, and survival packages (R Foundation for Statistical Computing, Vienna, Austria).

## RESULTS

### Stage 1: Evidence-based Mapping

The scoping review identified 63 studies including 28,860 patients. A PRISMA diagram of included studies is presented in Figure S1 (http://links.lww.com/AOSO/A138) and baseline characteristics are presented in Table S2 (http://links.lww.com/AOSO/A138). Most were retrospective cohort studies, and the overall chyle leak rate in these studies was 3.4% (949/28,500 patients) (Figure S2, http://links.lww.com/AOSO/A138). A summary of reported risk factors, intraoperative techniques and diagnosis of chyle leaks are presented in Table S3–S5 (http://links.lww.com/AOSO/A138).

#### Postoperative Management

**FIGURE 2.**
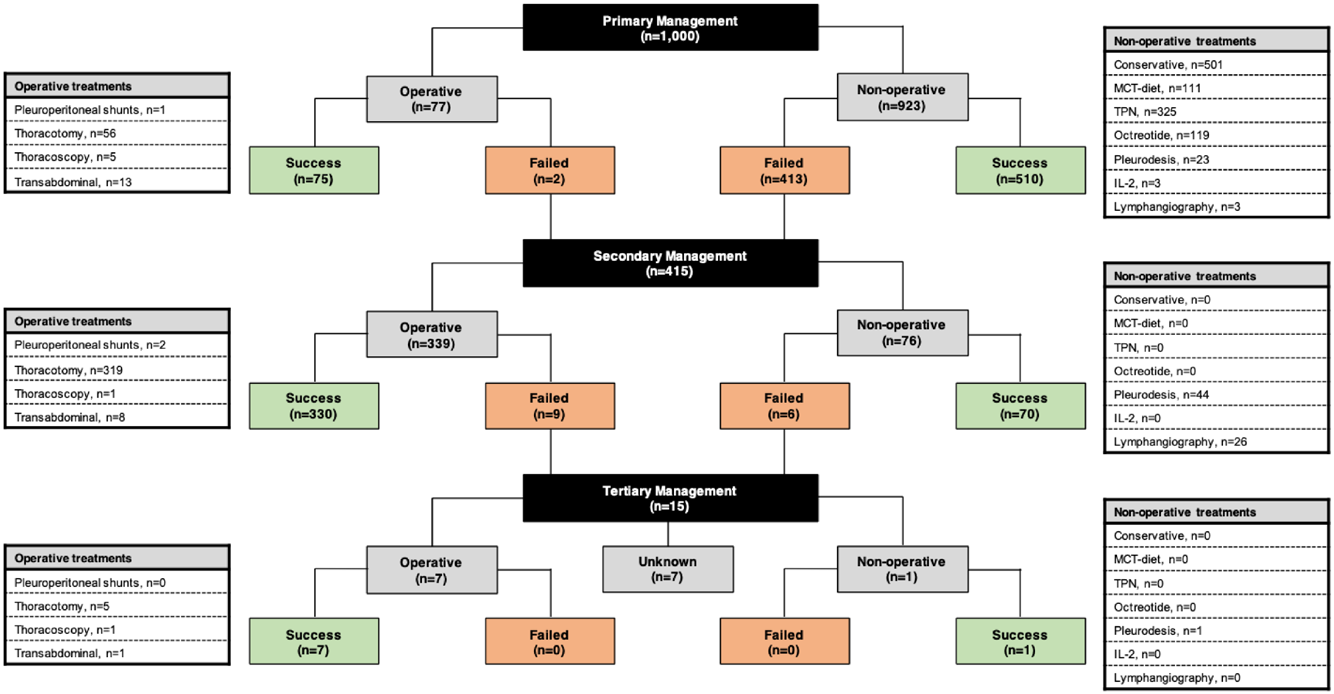
Flow chart describing primary, secondary and tertiary management of chyle leaks following esophagectomy for cancer from patient-level data from scoping review.

A flow chart summarizing management of chyle leaks is shown in Figure 2, which included 1,000 patients across 57 studies. The majority of patients (n = 923) received a nonoperative approach as their primary management, which was successful in 510 (55%) patients. Of the patients who required further management due to failure of an initial nonoperative approach (n = 415), the majority (339 patients, 81.7%) received surgery and 97% of them (330/339) had resolution of the chyle leak, compared with 92% (70/76) with nonoperative management (ie, pleurodesis (n = 44/48) and lymphangiography (n = 26/28). On the other hand, operative management when used as the primary treatment, was successful in 97% (75/77) of patients.

#### Summary

Based on themes of these studies, and initial discussions with the steering committee, a total of 41 different questions were developed. These topics were categorized into 5 broad domains: risk factors, intraoperative techniques, and postoperative management (ie, diagnosis, severity, and treatment). These thematic domains each incorporated several questions, which were agreed on by the steering committee before proceeding to the next stage.

### Stage 2: Impact of Chyle Leaks on Outcomes

Of the 2,247 patients identified from the OGAA study, 122 patients (5.4%) had a chyle leak, of which 26 (21.3%) and 54 (44.3%) patients developed type II and III chyle leaks, respectively. Baseline characteristics of patients developing chyle leaks are presented in Table S6 (http://links.lww.com/AOSO/A138). Across patient- and tumor-level characteristics, there were no significant differences in patients with and without a chyle leak. There were no significant differences in extent of nodal dissection on chyle leak rates. However, increasing severity of chyle leaks (type I vs type II vs type III) was associated with a significantly higher rates of pulmonary complications (31.0% vs 46.2% vs 53.7%, *P* < 0.001), higher rates of return to theater (9.5% vs 19.2% vs 77.8%, *P* < 0.001), longer length of stay (median: 23 vs 30 vs 34 days, *P* < 0.001), and higher 90-day mortality rates (0.0% vs 3.8% vs 14.8%, *P* < 0.001) (Table S7, http://links.lww.com/AOSO/A138).

### Stage 3: First Voting Round

During the first voting round, a total of 275 unique and complete responses were received by participants from 45 countries, the majority from Europe (175, 63.6%) and 163 (59.3%) were esophagogastric surgeons. Of the respondents, only 10 (3.6%) were high-volume surgeons (≥51 procedures) and 12 (4.4%) were from high-volume departments (≥101 procedures) (Table S8, http://links.lww.com/AOSO/A138). Results from the first round of voting are summarized in Tables S9 and S10 (http://links.lww.com/AOSO/A138). Overall, Cronbach’s α was 0.90 for agreement on each research question, suggesting good internal reliability.

### Stage 4: Second Voting Round

During the second round, a total of 250 unique and complete responses were received by participants from 43 countries. In this round, 165 (66.0%) were from Europe and 141 (56.4%) were oesophagogastric surgeons; only 11 (4.4%) were high-volume surgeons and 15 (6.0%) were from high-volume departments (Table S8, http://links.lww.com/AOSO/A138). Comparisons between stages 2 and 3 showed no significant differences in respondent characteristics (Table S8, http://links.lww.com/AOSO/A138) and the responses relating to research questions remained consistent between the 2 stages. Overall Cronbach’s α was 0.92 for agreement on each research question, suggesting good internal reliability. Thematic analyses of free-text responses across these different domains are summarized in Table S11 (http://links.lww.com/AOSO/A138).

#### Intraoperative Techniques

**FIGURE 3.**
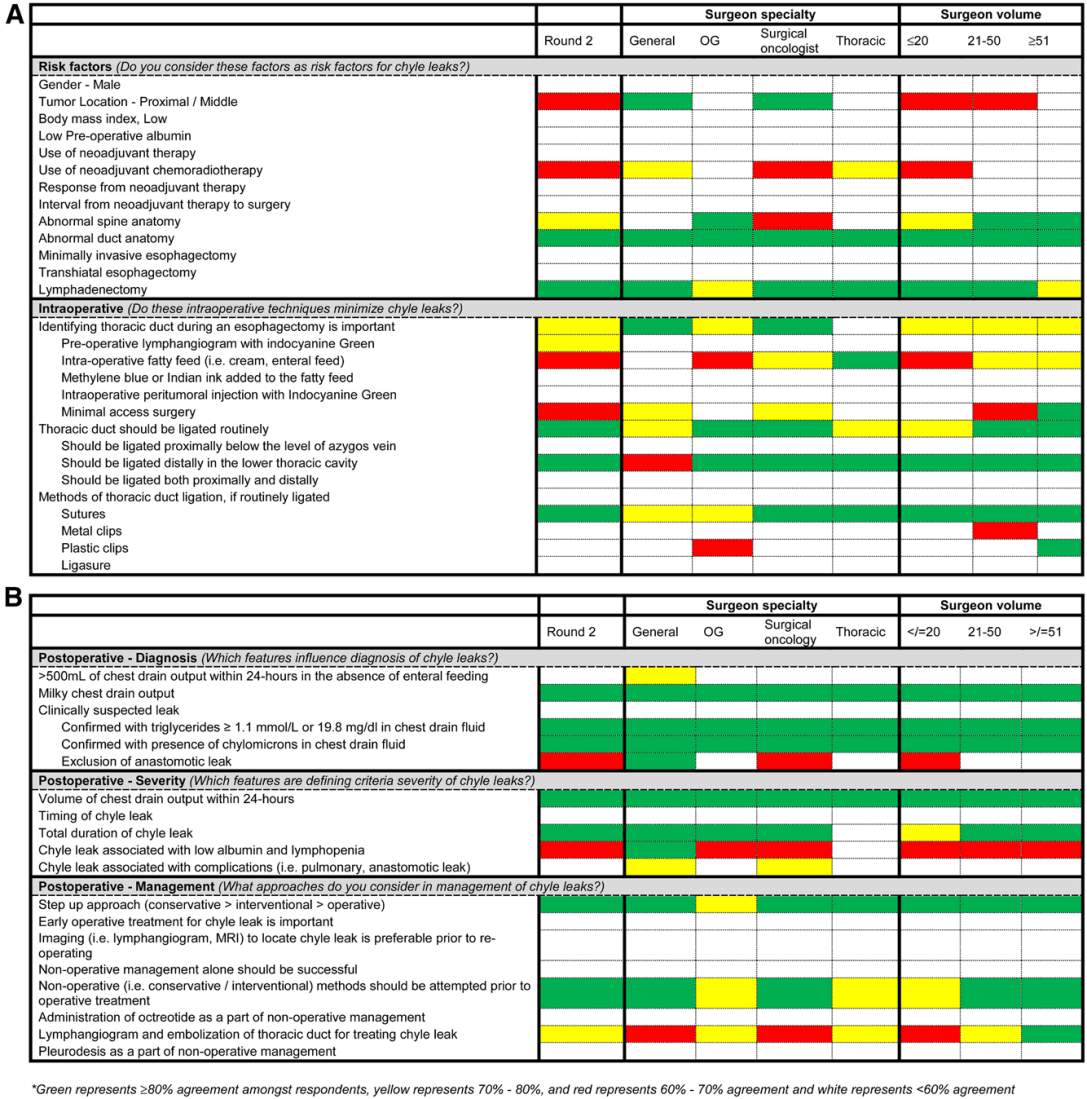
(A) Heat map of agreement of respondents on preoperative factors and intraoperative techniques from stage 3 of the modified Delphi exercise stratified by surgeon specialty and surgeon volume. (B) Heat map of agreement of respondents on postoperative factors and management of established chyle leak from stage 3 of the modified Delphi exercise stratified by surgeon specialty and surgeon volume. *Green represents ≥80% agreement among respondents, yellow represents 70%–80%, and red represents 60%–70% agreement and white represents <60% agreement.

Lymphadenectomy achieved agreement consensus as risk factors for chyle leaks, especially dissection around mediastinal and para-aortic nodes. However, consensus was not achieved by surgeon specialty or surgeon volume (Tables S12 and S13, http://links.lww.com/AOSO/A138). Heat map on the level of consensus across the different risk factors are presented in Figure 3A. Consensus agreement was achieved that thoracic duct should be routinely ligated at index operation (Table S9, http://links.lww.com/AOSO/A138). Consensus agreement was achieved in thoracic duct ligation distally in the lower thoracic cavity and ligation with sutures (Table S9, http://links.lww.com/AOSO/A138). Heat map on the level of consensus across the different intraoperative techniques are presented in Figure 3A.

#### Postoperative—Diagnosis

Although volume is used to assist in establishing diagnosis in the ECCG,^15^ consensus agreement was achieved for presence of milky chest drain output and presence of triglycerides and chylomicrons in chest drain output for diagnosis of chyle leaks (Table S10, http://links.lww.com/AOSO/A138). Stratified analysis by surgeon specialty and surgeon volume demonstrated consistent consensus agreement (Tables S12 and S13, http://links.lww.com/AOSO/A138). Heat map on the level of consensus across the different postoperative diagnosis are presented in Figure 3B.

#### Postoperative—Severity

Consensus agreement on severity of chyle leaks was achieved on the volume of chest (ie, >1L of chyle) drain output within 24 hours (Table S10, http://links.lww.com/AOSO/A138), which remained consistent across stratified analysis by surgeon specialty and surgeon volume (Tables S12 and S13, http://links.lww.com/AOSO/A138). Heat map on the level of consensus across the different postoperative diagnosis are presented in Figure 3B.

#### Postoperative—Management

Consensus agreement on management of chyle leaks were achieved for step-up approach and the use of nonoperative management prior to operative treatment (Table S10, http://links.lww.com/AOSO/A138). Heat map on the level of consensus across the different postoperative management are presented in Figure 3B.

### Stage 5: Recommendations for Clinical Practice

Focus groups with expert panel of esophageal surgeons were conducted, and data from scoping review, voting rounds, and the cohort study were presented for discussion. A summary of recommendations from the modified Delphi exercise is presented in Table 1.

**TABLE 1.** Summary of Recommendations From Consensus in the Management of Chyle Leaks Following Esophagectomy

**Preoperative**	
1.	Risk stratification to identify high-risk patients for chyle leaks should be considered
**Intraoperative**	
2.	Routine ligation of thoracic duct is recommended
**Postoperative**	
3.	Diagnosis of chyle leaks should be based on the following criteria:
	□ Excess volume (ie, >500 mL) of chest drain output within 24 hours
	□ Milky chest drain output
	□ Presence of triglycerides ≥ 1.1 mmol/L or 19.8 mg/dL in chest drain fluid
	□ Presence of chylomicrons in chest drain fluid
4.	Severity of chyle leaks should be assessed by:
	□ Volume of chest drain output AND/OR total duration of chyle leaks
5.	A step-up approach (conservative > interventional > operative) is recommended in the management of patients with chyle leaks
6.	Operative management should be considered once nonoperative options have not been successful

## DISCUSSION

This modified Delphi exercise has identified 5 broad themes on perioperative risk factors, intraoperative techniques, diagnosis, and severity of chyle leaks and management of chyle leaks following esophagectomy for cancer. Within these broad themes, consensus was reached by a diverse international group of esophageal surgeons. Variation in diagnosis and management of chyle leaks were also reported across surgeon and center volume. Developing pragmatic guidelines may allow prevention of, and timely diagnosis of chylothorax, and improve care of these patients.

Chyle leaks following esophagectomy are uncommon, thus risk factors associated with this complication remain unclear. Analysis from the OGAA data found advanced tumor stage and squamous cell carcinoma to be associated with chyle leaks.^14^ These findings may reflect wider dissection of tumor for advanced disease and as such increases risk for leaks when surgeons do not routinely ligate the duct. However, no consensus was achieved on the reported risk factors associated with chyle leaks, warranting a scrutiny of the quality of current evidence. First, wide anatomical variations of the thoracic duct^25^ has been thought to be associated with increased risk of chyle leaks.^4,26^ For instance, small leaks are thought to occur from lower thoracic duct tributaries that merge with the thoracic duct at the level of the diaphragm, which may be prevented through mass ligation of the surrounding connective tissue at the diaphragm. Second, a low body mass index has been demonstrated to increase risk of chyle leaks,^8,27,28^ although underlying mechanisms are not well established. Finally, neoadjuvant chemoradiotherapy (nCRT) were thought to be associated with higher chyle leak rates,^1,29,30^ in some observational studies. However, data from randomized trials have demonstrated similar rates of chyle leaks after nCRT compared to upfront surgery.^31,32^

Prophylactic ligation of the thoracic duct has been a topic of debate amongst esophageal surgeons, with some advocating for this as a routine in clinical practice while others do not. Dougenis et al^33^ demonstrated a significant reduction in the incidence of chyle leaks when routine ligation was undertaken (2.1% vs 9.0%), which have been supported in other series.^2,34^ Other studies suggest that the routine ligation of the thoracic duct may result in damage of the duct and thereby increase the risk of postoperative chyle leaks.^35,36^ A large database study (n = 12,237) from Japan showed that prophylactic thoracic duct ligation was associated with similar survival but higher rates of distant metastases in multiple organs in patients with esophageal cancer despite yielding a higher lymph node harvest and significantly fewer lymph node recurrence (376 vs 450, *P* = 0.003) than in patients where the thoracic duct was preserved.^36^ Recently, identification of the thoracic duct has been aided with adjuncts such as indocyanine green fluorescence, methylene blue, or preoperative oral ingestion of olive oil or cream.^37^ Indocyanine green fluorescence allows clear visualization of the main and accessory thoracic duct.^38^ Therefore, some authors consider it a good option to reduce the risk of chyle leaks after esophageal surgery. Our Delphi exercise indicates that surgeons consider extensive lymphadenectomy as a risk factor for chyle leaks after esophagectomy, consistent with findings from previous studies.^39^

To date, diagnostic criteria for chyle leaks following esophagectomy remain unclear and vary across published series.^2,40,41^ The recently published Esophageal Complications Consensus Group (ECCG) categorized the severity of chyle leak based on the treatment (ie, dietary, TPN, surgery and volume <1L or ≥1L 24 hours), with only 23% of patients with chyle leak requiring intervention or surgical treatment in their Esodata database.^15,42^ Our group reached consensus on supplementing diagnosis of chyle leaks with a focus on biochemical testing with presence of triglycerides and chylomicrons in the chest drain fluid, if required. Adopting defined criteria to establish diagnosis and assessment of severity of chyle leaks will allow standardization in clinical practice.

To date, management of chyle leaks remains highly variable with several treatment strategies reported. Dietary interventions, consisting of a low-fat diet with TPN aim to reduce chyle flow, allowing the chyle leak to obliterate.^35,43^ A systematic review revealed a clinical success rate ranging widely from 36.3% to 86.6% for conservative treatment,^10^ and addition of octreotide to standard nonoperative measures (chest drainage, TPN ± pleurodesis) increased the success rate with conservative treatment from 40% to 86.6%.^10^ Alternatively, others advocate early surgical intervention involving clipping or ligating the thoracic duct proximal to the leak.^34^ There is lack of agreement around the timing of reoperation—some surgeons propose several weeks of conservative (dietary) treatment before considering surgical management necessary,^1,43,44^ whereas others recommend early surgery.^8,45–47^ A recent study demonstrated that a step-up approach from nonoperative management with dietary measures controls a majority (up to 87%) of chyle leaks with a small number of refractory chyle leaks requiring surgery, consistent with the recommendations of our Delphi exercise.^48^ Newer interventions such as lymphangiography and embolization are increasingly used with good success rates and low morbidity.^10,41,49^

The main strength of this modified guideline consensus is the broad inclusion of specialist surgeons in the management of esophageal cancer across various settings, allowing generalizability of recommendations. Furthermore, we used robust mixed methods including scoping review, Delphi exercise, data from the OGAA international cohort study and focus groups. However, there are limitations to be recognized. First, most recommendations are made from expert consensus based on limited evidence from the literature. Second, the OGAA cohort study did not include data on the specific risk factors, diagnosis, or management (ie, thoracic duct ligation) of chyle leaks, which would have been useful in understanding a global approach in the management of chyle leaks.

## CONCLUSION

In summary, we reached consensus on possible perioperative risk factors, intraoperative techniques to decrease the incidence, diagnostic criteria, and management of chyle leaks following esophagectomy for cancer. Prospective standardized use of these definitions and recommendations, combined with documentation in large databases such as the EsoData and OGAA will improve the quality and quantity of data, and advance knowledge in this under-researched area.

**FIGURE 4.**
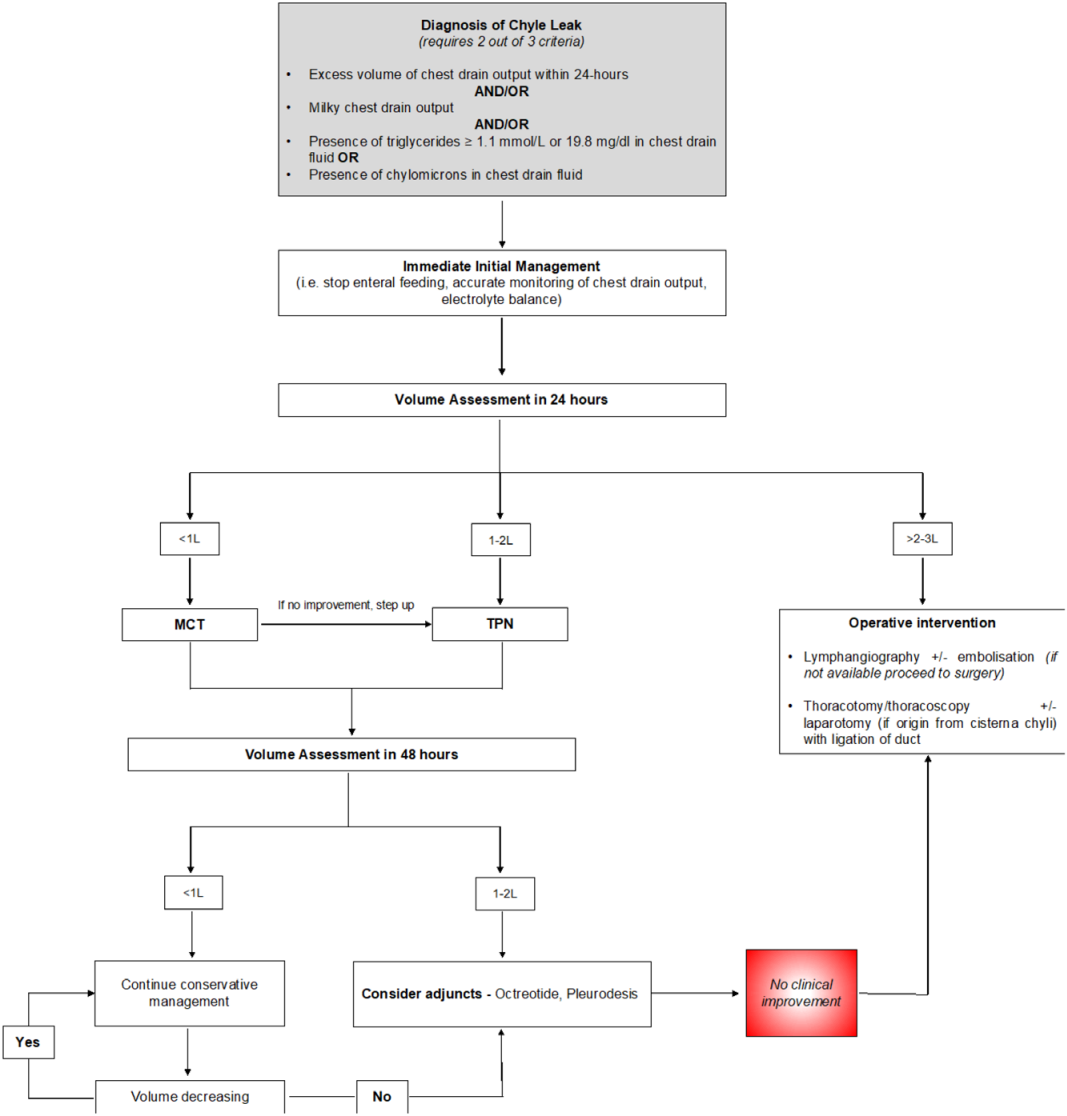
Proposed algorithm in the diagnosis and management of chyle leaks after esophagectomy for cancer.

## Supplementary Material


