# Supplementary material for: Risk Factors, Diagnosis and Management of Chyle Leak Following Esophagectomy for Cancers: An International Consensus Statement
Source: Ann Surg Open. 2022 Aug 29;3(3):e192. doi: 10.1097/AS9.0000000000000192 (PMC9508983; doi:10.1097/AS9.0000000000000192)

Supplementary Table 1 Summary of search terms used for systematic review

	Name	Number
1	esophagectomy.ti,ab.	10246
2	oesophagectomy.ti,ab.	1597
3	oesophagogastrectomy.ti,ab.	94
4	esophagogastrectomy.ti,ab.	520
5	8 or 9 or 10 or 11	12255
6	management.ti,ab.	1207844
7	treatment.ti,ab.	4588649
8	risk factors.ti,ab.	480929
9	diagnosis.ti,ab.	1616129
10	1 or 2 or 17 or 18	6713911
11	chylothorax.ti,ab.	3556
12	chyle leak.ti,ab.	239
13	exp Chylothorax/	3126
14	chyle leakage.ti,ab.	159
15	chylous fistula.ti,ab.	142
16	4 or 5 or 6 or 14 or 20	4568
17	12 and 19 and 21	164

Supplementary Table 2 Baseline characteristics of included studies into the scoping review

Study Name	Study Period	Study Type	Patients, n	Chyle Leak, n (%)
**Cohort study**			28500	949 (3.3)
Orringer 1988	1988	Retrospective	320	11 (3.4)
Alexiou 1998	Jan 1987 - Nov 1997	Retrospective	523	21 (4.0)
Dugue 1998	1980 - 1996	Retrospective	850	23 (2.7)
Bolger 1991	1977 - 1990	Retrospective	537	11 (2.0)
Dougenis 1992	Jan 1983 - May 1987	Retrospective	255	10 (3.9)
Merigliano 2000	1980 - 1998	Retrospective	1787	19 (1.1)
Bonavina 2001	Nov 1992 - Dec 2000	Retrospective	316	3 (0.9)
Lagarde 2005	Jan 1995 - Dec 2003	Retrospective	536	20 (3.7)
Hayden 2007	Jun 2002 - Aug 2005	Retrospective	129	6 (4.7)
Schumacher 2007	1998 - 2005	Retrospective	409	10 (2.4)
Benedix 2010	Dec 2006 - Nov 2008	Retrospective	56	3 (5.4)
Lai 2011	Jul 2004 - Jun 2009	Retrospective	653	8 (1.2)
Guo 2012	May 2009 - Jun 2010	Retrospective	135	8 (5.9)
Shah 2012	Jan 1997 - Jul 2008	Retrospective	892	34 (3.8)
Li 2013	Jan 1996 - Dec 2011	Retrospective	10574	306 (2.9)
Mishra 2013	Oct 2003 - Jul 2011	Retrospective	104	9 (8.7)
Fujita 2014	2001 - 2009	Retrospective	521	20 (3.8)
Hou 2014	Dec 1996 - Dec 2008	Retrospective	1804	17 (0.9)
Kim 2014	Sep 1994 - Dec 2010	Retrospective	1514	57 (3.8)
Gupta 2015	Jan 2010 - Jul 2012	Retrospective	45	4 (8.9)
Miao 2015	Feb 2007 - Dec 2012	Retrospective	1290	34 (2.6)
Brinkmann 2016	Jan 2005 - Dec 2013	Retrospective	906	17 (1.9)
Lin 2017	March 2011 - Dec 2015	Retrospective	296	5 (1.7)
Weijs 2017	Oct 2003 - May 2014	Retrospective	371	78 (21.0)
Oguma 2018	Aug 2014 - Apr 2017	Retrospective	290	11 (3.8)
Ohkura 2018	Jan 2011 - Feb 2017	Retrospective	371	19 (5.1)
Ohkura 2018	Jan 2011 - June 2017	Retrospective	294	24 (8.2)
Batool 2019	Jan 2009 - Dec 2016	Retrospective	193	9 (4.7)
Schurink 2019	Jan 2012 - Dec 2017	Retrospective	198	44 (22.2)
Malibary 2020	Jan 2013 - Dec 2016	Retrospective	70	7 (10.0)
Farran 2021	Jan 2010 - Apr 2019	Retrospective	274	13 (4.7)
Higuchi 2021	Sept 2009 - Feb 2019	Retrospective	390	12 (3.1)
Jeon 2021	Jan 2015 - Jun 2017	Retrospective	605	26 (4.3)
Milito 2021	Jan 1997 - Dec 2017	Retrospective	992	50 (5.0)
**Case series**			140	140 (100.0)
Guillem 1999	1999	Retrospective	3	3 (100.0)
Omloo 2006	2006	Retrospective	2	2 (100.0)
Chen 2010	2010	Retrospective	5	5 (100.0)
Marthaller 2015	Jun 2008 - Dec 2012	Retrospective	5	5 (100.0)
Ohkura 2015	2015	Retrospective	2	2 (100.0)
Liu 2016	2016	Retrospective	3	3 (100.0)
Alamdari 2018	Mar 2009 - Apr 2014	Retrospective	98	98 (100.0)
Lambertz 2019	-	Retrospective	4	4 (100.0)
Jardinet 2021	May 2015 - Mar 2019	Retrospective	18	18 (100.0)

**Case report**			20	20 (100.0)
Nakano 1994	1994	-	1	1 (100.0)
Fujiwara 1999	1999	-	1	1 (100.0)
Bybel 2001	2001	-	1	1 (100.0)
Motoyama 2005	2004	-	1	1 (100.0)
Nadesan 2005	2005	-	1	1 (100.0)
Ginat 2009	2009	-	1	1 (100.0)
Windhaber 2010	2010	-	1	1 (100.0)
Rottoli 2012	2012	-	1	1 (100.0)
Matsutani 2014	2014	-	1	1 (100.0)
Yamamoto 2015	2015	-	1	1 (100.0)
Atie 2016	2016	-	1	1 (100.0)
Chang 2017	Sep 2016	-	1	1 (100.0)
Shimakawa 2017	2017	-	1	1 (100.0)
Ishida 2019	Nov 2017	-	1	1 (100.0)
Taki 2019	Jan 2019	-	1	1 (100.0)
Drabkin 2020	Jul 2020	-	1	1 (100.0)
Haneda 2020	Mar 2020	-	1	1 (100.0)
Sesti 2020	Aug 2019	-	1	1 (100.0)
Suetsugu 2020	Apr 2020	-	1	1 (100.0)
Sato 2021	May 2021	-	1	1 (100.0)

Supplementary Table 3 Summary of reported preoperative, intraoperative, and postoperative risk factors for chyle leak in multivariate analysis following esophagectomy for cancer from scoping review

	**Surgeon-level**	**Preoperative**					**Intraoperative**			**Postoperative**
Study Name	Junior Surgeon	BMI, kg/m^2	CCI	Tumour Location	Tumor Histology	Neoadjuvant Therapy	Surgical Approach	Adhesions	Intraoperative Fluid Balance	Anastomotic Leak
Bolger 1991		NS					-			
Alexious 1998		NS					-			
Lagarde 2005							Transthoracic			
Shah 2012		<30	≥2		SCC					
Gupta 2015				NS		nCT or nCRT				
Miao 2015		<25								
Brinkmann 2016										
Weijs 2017		NS				nCT or nCRT	Transthoracic			
Ohkura 2018						nCRT			High	
Batool 2019								Yes		
Schurink 2019		NS				nCT or nCRT	Transhiatal			Yes
Malibary 2020	Yes									

**Abbreviations: BMI: body mass index, CCI: Charlson Comorbidity Index, nCRT: neoadjuvant chemoradiotherapy, nCT: neoadjuvant chemotherapy, NS: not stated, SCC: squamous cell carcinoma*

Supplementary Table 4 Summary of reported intraoperative techniques for thoracic duct ligation on reducing chyle leak following esophagectomy for cancer from scoping review

Study Name	Ligation or Resection of Duct	Approach
Dougenis 1992	Selective	Ligation
Dugue 1998	Routine	Ligation
Bonavina 2001	Routine	Ligation
Lagarde 2005	Selective	Ligation
Motoyama 2005	None	N/A
Omloo 2006	Routine	Ligation
Hayden 2007	Selective	Ligation
Lai 2011	Selective	Ligation
Guo 2012	Selective	Ligation
Rottoli 2012	Routine	Ligation
Fujita 2014	Selective	Resection
Hou 2014	Selective	Ligation
Matsutani 2014	Routine	Resection
Gupta 2015	None	N/A
Miao 2015	Routine	Ligation
Ohkura 2015	Routine	Resection
Yamamoto 2015	Routine	Ligation
Atie 2016	Routine	Resection
Brinkmann 2016	Routine	Ligation
Chang 2017	Routine	Ligation
Lin 2017	Selective	Ligation
Weijs 2017	Routine	Ligation
Oguma 2018	Routine	Ligation
Ohkura 2018	Routine	Ligation
Batool 2019	Routine	Resection
Ishida 2019	Routine	Ligation
Schurink 2019	None	N/A
Taki 2019	None	N/A
Haneda 2020	Routine	Resection
Malibary 2020	Routine	Ligation
Sesti 2020	Routine	Ligation
Suetsugu 2020	None	N/A
Higuchi 2021	Routine	Ligation
Jardinet 2021	Selective	Ligation
Milito 2021	Routine	Ligation

Supplementary Table 5 Summary of reported definitions used for diagnosis of chyle leaks following esophagectomy for cancer from scoping review

	Criteria for Diagnosis				
**Study Name**	**Volume**	**Volume, mL**	**Colour**	**Chylomicron**	**Triglyceride**
Orringer 1988	Yes	NR	Yes	-	-
Bolger 1991	Yes	NR	Yes	-	-
Nakano 1994	Yes	900	Yes	-	-
Alexiou 1998	Yes	1,000	Yes	Yes	Yes
Dugue 1998	Yes	500	-	-	-
Fujiwara 1999	Yes	1,600	-	-	-
Guillem 1999	Yes	300	-	-	-
Merigliano 2000	Yes	1,000	Yes	-	Yes
Bonavina 2001	-	-	Yes	-	-
Lagarde 2005	-	-	Yes	-	Yes
Motoyama 2005	Yes	1,000	Yes	-	Yes
Nadesan 2005	-	-	Yes	-	-
Omloo 2006	Yes	2,500	Yes	-	-
Hayden 2007	Yes	1,000	-	-	-
Schumacher 2007	-	-	Yes	-	Yes
Ginat 2009	-	-	-	-	Yes
Benedix 2010	-	-	-	Yes	Yes
Chen 2010	-	-	-	-	Yes
Windhaber 2010	-	-	-	Yes	-
Lai 2011	Yes	1,000	Yes	-	-
Rottoli 2012	-	-	-	-	Yes
Shah 2012	Yes	NR	Yes	Yes	Yes
Li 2013	Yes	500	Yes	-	Yes
Mishra 2013	-	-	-	-	Yes
Fujita 2014	Yes	1,000	Yes	-	-
Matsutani 2014	Yes	1,500	-	-	-
Gupta 2015	-	-	-	-	Yes
Miao 2015	-	-	Yes	Yes	-
Ohkura 2015	Yes	2,000	Yes	-	-
Yamamoto 2015	-	-	Yes	-	-
Atie 2016	Yes	200	Yes	-	-
Brinkmann 2016	Yes	1,000	Yes	-	-
Liu 2016	-	-	Yes	-	-
Chang 2017	-	-	-	-	Yes
Lin 2017	-	-	-	-	Yes
Shimakawa 2017	Yes	1,000	-	-	-
Weijs 2017	-	-	-	-	Yes
Ohkura 2018	-	-	-	-	Yes
Ohkura 2018	Yes	800	Yes	Yes	Yes
Batool 2019	Yes	NR	Yes	-	Yes
Ishida 2019	Yes	1,000	-	-	-
Lambertz 2019	-	-	-	-	-
Schurink 2019	-	-	-	-	-

Taki 2019	Yes	600	Yes	-	Yes
Haneda 2020	Yes	NR	-	-	-
Malibary 2020	-	-	-	-	-
Sesti 2020	Yes	2,500	Yes	-	-
Suetsugu 2020	Yes	1,000	Yes	-	-
Milito 2021	Yes	500	Yes	Yes	Yes
Sato 2021	Yes	4,000	-	-	-

Supplementary Table 6 Clinicopathologic characteristics of patients with and without chyle leaks undergoing esophagectomy for esophageal cancers from the Oesophagogastric Anastomosis Audit

		**No, 2,125**	**Yes, n=122**	**p-value**
**Center-level factors**				
Center volume	<28	712 (33.5)	33 (27.0)	0.3
	28-50	698 (32.8)	47 (38.5)	
	>50	715 (33.6)	42 (34.4)	
Country income	HIC	1836 (86.4)	103 (84.4)	0.6
	LMIC	289 (13.6)	19 (15.6)	
**Patient-level factors**				
Age, years	Mean (SD)	64.0 (10.5)	62.3 (10.6)	0.1
Gender	Female	445 (20.9)	35 (28.7)	0.055
	Male	1680 (79.1)	87 (71.3)	
ASA Grade	1	284 (13.4)	15 (12.3)	0.1
	2	1180 (55.5)	80 (65.6)	
	3	639 (30.1)	27 (22.1)	
	4	22 (1.0)	0 (0.0)	
Smoking status	Never	793 (37.3)	49 (40.2)	0.8
	Ex	952 (44.8)	49 (40.2)	
	Current	320 (15.1)	20 (16.4)	
	Unknown	60 (2.8)	4 (3.3)	
Body mass index, kg/m^2	<18.5	95 (4.5)	7 (5.7)	0.2
	18.6-24.9	840 (39.5)	54 (44.3)	
	25.0-29.9	758 (35.7)	32 (26.2)	
	>30	432 (20.3)	29 (23.8)	
Tumor type	Adenocarcinoma	1573 (74.0)	80 (65.6)	0.049
	Squamous cell carcinoma	494 (23.2)	40 (32.8)	
	Other	58 (2.7)	2 (1.6)	
Tumor location	Proximal/Middle	273 (12.8)	25 (20.5)	0.065
	Distal	694 (32.7)	39 (32.0)	
	Siewert 1	632 (29.7)	36 (29.5)	
	Siewert 2-3	525 (24.7)	22 (18.0)	
	(Missing)	1 (0.0)	0 (0.0)	
AJCC Clinical T stage	cT0/Tx	97 (4.6)	3 (2.5)	0.026
	cT1	168 (7.9)	6 (4.9)	
	cT2	453 (21.3)	14 (11.5)	
	cT3	1299 (61.1)	92 (75.4)	
	cT4a	102 (4.8)	6 (4.9)	
	cT4b	6 (0.3)	1 (0.8)	
AJCC Clinical N stage	cN0/Nx	940 (44.2)	47 (38.5)	0.6
	cN1	807 (38.0)	53 (43.4)	
	cN2	318 (15.0)	18 (14.8)	
	cN3	60 (2.8)	4 (3.3)	
Preoperative nutrition	None	1082 (50.9)	45 (36.9)	0.022
	Oral Supplements	777 (36.6)	59 (48.4)	
	Enteral Nutrition	233 (11.0)	15 (12.3)	
	Parenteral Nutrition	33 (1.6)	3 (2.5)	

Surgical approach	Open	820 (38.6)	55 (45.1)	0.4
	Hybrid	556 (26.2)	32 (26.2)	
	MIO	627 (29.5)	31 (25.4)	
	Transhiatal	122 (5.7)	4 (3.3)	
Anastomosis site	Chest at Azygous	695 (32.7)	34 (27.9)	0.1
	Chest below Azygous	267 (12.6)	8 (6.6)	
	Chest above Azygous	678 (31.9)	48 (39.3)	
	Neck	485 (22.8)	32 (26.2)	
AJCC Pathological T stage	pT0/Tx	361 (17.0)	21 (17.2)	0.9
	pT1a	138 (6.5)	6 (4.9)	
	pT1b	277 (13.0)	12 (9.8)	
	pT2	301 (14.2)	18 (14.8)	
	pT3	964 (45.4)	60 (49.2)	
	pT4a	79 (3.7)	5 (4.1)	
	pT4b	5 (0.2)	0 (0.0)	
AJCC Pathological N stage	pN0	1133 (53.3)	63 (51.6)	0.8
	pN1	551 (25.9)	34 (27.9)	
	pN2	187 (8.8)	9 (7.4)	
	pN3	240 (11.3)	16 (13.1)	
	(Missing)	14 (0.7)	0 (0.0)	
AJCC Pathological Overall stage	Stage 0	296 (14.8)	17 (15.0)	0.6
	Stage IA	119 (5.9)	5 (4.4)	
	Stage IB	192 (9.6)	8 (7.1)	
	Stage IC	113 (5.6)	5 (4.4)	
	Stage IIA	77 (3.8)	9 (8.0)	
	Stage IIB	331 (16.5)	16 (14.2)	
	Stage IIIA	100 (5.0)	4 (3.5)	
	Stage IIIB	490 (24.5)	30 (26.5)	
	Stage IVA	243 (12.1)	16 (14.2)	
	Stage IVB	42 (2.1)	3 (2.7)	
Margin status	R0	1734 (81.6)	105 (86.1)	0.3
	R1	391 (18.4)	17 (13.9)	
Lymph nodes examined	Mean (SD)	24.4 (12.8)	25.9 (13.8)	0.2

Supplementary Table 7 Impact of grade of chyle leaks on post-operative outcomes in patients undergoing esophagectomy for cancer from the OGAA cohort

	**None**	**Grade A**	**Grade B**	**Grade C**	**p-value**
Overall complications	1310 (61.6)	40 (95.2)	25 (96.2)	54 (100.0)	**<0.001**
Major complications	495 (23.3)	12 (28.6)	15 (57.7)	50 (92.6)	**<0.001**
Conduit Necrosis	56 (2.6)	1 (2.4)	1 (3.8)	2 (3.7)	0.9
Postoperative pulmonary complications	752 (35.4)	13 (31.0)	12 (46.2)	29 (53.7)	**0.025**
Return to theatre	218 (10.3)	4 (9.5)	5 (19.2)	42 (77.8)	**<0.001**
Total ICU stay, days	5.6 (8.6)	8.8 (13.3)	7.0 (7.4)	11.3 (15.1)	**<0.001**
Total LOS, days	16.3 (13.6)	23.1 (17.9)	30.3 (20.8)	34.1 (19.7)	**<0.001**
Eating on discharge	1866 (87.8)	39 (92.9)	16 (61.5)	46 (85.2)	**0.001**
30-day readmission	228 (10.7)	6 (14.3)	4 (15.4)	11 (20.4)	0.1
90-day mortality	91 (4.3)	0 (0.0)	1 (3.8)	8 (14.8)	**0.001**

**Grading of chyle leak was based on the Esophageal Complications Consensus Group (ECCG), which are type I (requiring enteral dietary modification), type II (requiring TPN) and type III (requiring interventional or surgical treatment). Further division of each grade is possible based on output volume (i.e. type A with <1 L daily output and type B with >1 L daily output).*

Supplementary Table 8 Baseline characteristics and routine clinical practice of respondents from stage 2 and 3 of the modified Delphi consensus processes

		**Round 1, n=275**	**Round 2, n=250**	**p-value**
**Baseline characteristics**				
Age	Median (IQR)	46.0 (40.5 - 53.0)	46.0 (40.0 - 53.0)	0.942
Gender	Female	24 (8.7)	25 (10.0)	0.726
	Male	251 (91.3)	225 (90.0)	
Specialty	General Surgery	38 (13.8)	46 (18.4)	0.469
	Oesophagogastric Surgery	163 (59.3)	141 (56.4)	
	Surgical oncology	32 (11.6)	31 (12.4)	
	Thoracic Surgery	42 (15.3)	32 (12.8)	
Hospital Type	Regional/ county/ district general	31 (11.3)	29 (11.6)	1.000
	Tertiary/ Quaternary	244 (88.7)	221 (88.4)	
Department Volume, cases/year	≤50	172 (62.5)	175 (70.0)	0.062
	51-100	91 (33.1)	60 (24.0)	
	≥101	12 (4.4)	15 (6.0)	
Surgeon Volume	≤20	183 (66.5)	177 (70.8)	0.419
	21-50	82 (29.8)	62 (24.8)	
	≥51	10 (3.6)	11 (4.4)	
Time as consultant / attending, years	Median (IQR)	11.0 (6.0 to 20.0)	11.0 (6.0 to 20.0)	0.663
Country income	High	222 (80.7)	195 (78.0)	0.869
	Middle	51 (18.6)	53 (21.2)	
	Low	2 (0.7)	2 (0.8)	
**Non-operative adjuncts**				
Total parenteral nutrition	Yes	262 (95.3)	237 (94.8)	0.962
Medium chain triglycerides	Yes	244 (88.7)	213 (85.2)	0.284
Lymphangiogram	Yes	179 (65.1)	158 (63.2)	0.719
Pleurodesis	Yes	192 (69.8)	184 (73.6)	0.388
**Pleurodesis** - *as adjuncts to routine management*				
Talc	Yes	119 (43.3)	116 (46.4)	0.528
Tetracycline	Yes	41 (14.9)	34 (13.6)	0.762
Fibrin	Yes	27 (9.8)	28 (11.2)	0.709
Glucose	Yes	5 (1.8)	7 (2.8)	0.646
Bleomycin	Yes	21 (7.6)	21 (8.4)	0.872
Blood Patch	Yes	15 (5.5)	15 (6.0)	0.936
**Operative management**				
Surgical Ligations	Yes	242 (88.0)	217 (86.8)	0.778
Clips	Yes	209 (76.0)	187 (74.8)	0.828
Blood Patch	Yes	209 (76.0)	187 (74.8)	0.828
Glue	Yes	51 (18.5)	51 (20.4)	0.670

Supplementary Table 9 Summary of agreement of respondents from stage 2 and 3 of the modified Delphi exercise across the preoperative factors and intraoperative techniques domains

	**Round 1, n=275**	**Round 2, n=250**
**Risk factors** *(Do you consider these factors as risk factors for chyle leaks?)*		
Gender - Male	38 (13.8)	46 (18.4)
Tumor Location - Proximal / Middle	132 (48.0)	168 (67.2)
Body mass index, Low	84 (30.5)	81 (32.4)
Low pre-operative albumin	73 (26.5)	68 (27.2)
Use of neoadjuvant therapy	136 (49.5)	61 (24.4)
Use of neoadjuvant chemoradiotherapy*	-	156 (62.4)
Response from neoadjuvant therapy	98 (35.6)	97 (38.8)
Interval from neoadjuvant therapy to surgery	67 (24.4)	57 (22.8)
Abnormal spinal anatomy	163 (59.3)	185 (74.0)
Abnormal duct anatomy	240 (87.3)	234 (93.6)
Minimally invasive esophagectomy	30 (10.9)	23 (9.2)
Transhiatal esophagectomy	102 (37.1)	96 (38.4)
Lymphadenectomy	209 (76.0)	205 (82.0)
**Intraoperative** *(Do these intraoperative techniques minimize chyle leaks?)*		
Identifying thoracic duct during an esophagectomy is important	201 (73.1)	188 (75.2)
Pre-operative lymphangiogram with indocyanine Green	199 (72.4)	195 (78.0)
Intra-operative fatty feed (i.e. cream, enteral feed)	155 (56.4)	172 (68.8)
Methylene blue or Indian ink added to the fatty feed	68 (24.7)	44 (17.6)
Intraoperative peritumoural injection with Indocyanine Green	35 (12.7)	27 (10.8)
Minimal access surgery	147 (53.5)	150 (60.0)
Thoracic duct should be ligated routinely	213 (77.5)	201 (80.4)
Should be ligated proximally below the level of azygos vein	87 (31.6)	82 (32.8)
Should be ligated distally in the lower thoracic cavity	213 (77.5)	211 (84.4)
Should be ligated both proximally and distally	93 (33.8)	67 (26.8)
Methods of thoracic duct ligation, if routinely ligated		
Sutures	218 (79.3)	203 (81.2)
Metal clips	161 (58.5)	142 (56.8)
Plastic clips	154 (56.0)	135 (54.0)
Ligasure	25 (9.1)	19 (7.6)

**This question was introduced during the second round of voting*

Supplementary Table 10 Summary of agreement of respondents from stage 2 and 3 of the modified Delphi exercise across the postoperative diagnosis and management of chyle leaks domains

	**Round 1, n=275**	**Round 2, n=250**
**Postoperative - Diagnosis** *(Which features influence diagnosis of chyle leaks?)*		
>500mL of chest drain output within 24-hours in the absence of enteral feeding	126 (45.8)	135 (54.0)
Milky chest drain output	234 (85.1)	232 (92.8)
Clinically suspected leak		
Confirmed with triglycerides ≥ 1.1 mmol/L or 19.8 mg/dl in chest drain fluid	219 (79.6)	219 (87.6)
Confirmed with presence of chylomicrons in chest drain fluid	230 (83.6)	221 (88.4)
Exclusion of anastomotic leak	156 (56.7)	155 (62.0)
**Postoperative - Severity** *(Which features are defining criteria severity of chyle leaks?)*		
Volume of chest drain output within 24-hours*	242 (88.0)	234 (93.6)
Timing of chyle leak	98 (35.6)	77 (30.8)
Total duration of chyle leak (i.e. <48 hours or ≥48 hours)	177 (64.4)	200 (80.0)
Chyle leak associated with low albumin and lymphopenia	158 (57.5)	168 (67.2)
Chyle leak associated with complications (i.e. pulmonary, anastomotic leak)	119 (43.3)	133 (53.2)
**Postoperative - Management** *(What approaches do you consider in management of chyle leaks?)*		
Step up approach (conservative > interventional > operative)	-	207 (82.8)
Early operative treatment for chyle leak is important	127 (46.2)	94 (37.6)
Imaging (i.e lymphangiogram, MRI) to locate chyle leak is preferable prior to re-operating	134 (48.7)	128 (51.2)
Non-operative management alone should be successful	115 (41.8)	110 (44.0)
Non-operative (i.e. conservative / interventional) methods should be attempted prior to operative treatment	-	200 (80.0)
Administration of octreotide as a part of non-operative management	97 (35.3)	84 (33.6)
Lymphangiogram and embolization of thoracic duct for treating chyle leak	178 (64.7)	175 (70.0)
Pleurodesis as a part of non-operative management	56 (20.4)	43 (17.2)

**Volumes used to define severity of chyle leak was a 1 litre cut-off as used in both rounds of the chyle leak*

Supplementary Table 11 Thematic analyses of free text responses from stage 2 and 3 of voting across the five domains of risk factors, intraoperative techniques, and postoperative diagnosis and management of chyle leaks

	Number of respondents, n
**Risk factors** *(Do you consider these factors as risk factors for chyle leaks?)*	
Surgeon experience/volume	13
Advanced clinical T and N stage	10
Size of tumour	8
Resection / ligation of thoracic duct	7
Previous mediastinum/thoracic surgery	5
Level of the tumor (i.e. lower esophageal)	4
High body mass index/Obesity	3
High-risk patients (i.e. liver cirrhosis)	3
Tumor histology SCC	3
Definitive radiation	3
Anatomy variations	2
Others* (Level of thoracic duct ligation, Lymphoma, intraoperative bleeding, preoperative nutrition, smoking)	1
**Intraoperative** *(Do these intraoperative techniques minimize chyle leaks?)*	
Knowledge of anatomy	6
Ligation/clipping proximally/distally to help identifying by distension	3
Robotic approach	2
Dissection near oesophageal wall	2
Use of adjuncts (patent blue V, Indocyanine green)	2
Dissection of the duct	1
Preoperative lymphoangiography	1
**Postoperative - Severity** *(Which features are defining criteria severity of chyle leaks?)*	
Volume (< 500, >1000ml)	4
ECCG definition and Clavien Dindo score	3
Enteral feeding	2
No resolution (i.e. weight loss) with conservative management (TPN or MCT feed)	2
Preoperative albumin levels	1
**Postoperative - Management** *(What approaches you consider in management of chyle leaks?)*	
Step up approach MDT discussion (includes conservative management for small leaks)	6
Early reoperation/intervention depending on volume	3
Reoperation with approach to cisterna chyli	2
Pleuroperitoneal shunt	2
Pleurectomy	1
Pleurodesis	1
Interventional radiology	1
Transhiatal ligation1	1
Reduced fat diet	1

Supplementary Table 12 Summary of agreement of respondents from 3 of the modified Delphi process across the risk factors, intraoperative techniques, and postoperative diagnosis and management of chyle leaks domains stratified by specialty type

	**General, n=46**	**OG, n=141**	**Surgical Oncology, n=31**	**Thoracic, n=32**	**p-value**
**Risk factors** *(Do you consider these factors as risk factors for chyle leaks?)*					
Gender - Male	15 (32.6)	21 (14.9)	8 (25.8)	2 (6.2)	0.009
Tumor Location - Proximal / Middle	40 (87.0)	79 (56.0)	27 (87.1)	22 (68.8)	<0.001
Body mass index, Low	15 (32.6)	47 (33.3)	10 (32.3)	9 (28.1)	1.0
Low pre-operative albumin	21 (45.7)	27 (19.1)	13 (41.9)	7 (21.9)	0.001
Use of neoadjuvant therapy	18 (39.1)	24 (17.0)	10 (32.3)	9 (28.1)	0.013
Use of neoadjuvant chemoradiotherapy*	34 (73.9)	79 (56.0)	19 (61.3)	24 (75.0)	0.065
Response from neoadjuvant therapy	24 (52.2)	46 (32.6)	13 (41.9)	14 (43.8)	0.103
Interval from neoadjuvant therapy to surgery	19 (41.3)	22 (15.6)	6 (19.4)	10 (31.2)	0.002
Abnormal spinal anatomy	30 (65.2)	117 (83.0)	20 (64.5)	18 (56.2)	0.002
Abnormal duct anatomy	40 (87.0)	138 (97.9)	27 (87.1)	29 (90.6)	0.016
Minimally invasive esophagectomy	5 (10.9)	11 (7.8)	5 (16.1)	2 (6.2)	0.458
Transhiatal esophagectomy	18 (39.1)	54 (38.3)	10 (32.3)	14 (43.8)	0.8
Lymphadenectomy	37 (80.4)	110 (78.0)	29 (93.5)	29 (90.6)	0.111
**Intraoperative** *(Do these intraoperative techniques minimize chyle leaks?)*					
Identifying thoracic duct during an esophagectomy is important					
Pre-operative lymphangiogram with indocyanine Green	38 (82.6)	112 (79.4)	27 (87.1)	11 (34.4)	<0.001
Intra-operative fatty feed (i.e. cream, enteral feed)	18 (39.1)	24 (17.0)	6 (19.4)	6 (18.8)	0.009
Methylene blue or Indian ink added to the fatty feed	26 (56.5)	96 (68.1)	23 (74.2)	27 (84.4)	0.018
Intraoperative peritumoural injection with Indocyanine Green	16 (34.8)	17 (12.1)	7 (22.6)	4 (12.5)	0.003
Minimal access surgery	7 (15.2)	14 (9.9)	4 (12.9)	2 (6.2)	0.196
Thoracic duct should be ligated routinely	33 (71.7)	82 (58.2)	23 (74.2)	12 (37.5)	0.006
Should be ligated proximally below the level of azygos vein	35 (76.1)	118 (83.7)	25 (80.6)	23 (71.9)	0.394
Should be ligated distally in the lower thoracic cavity	17 (37.0)	45 (31.9)	9 (29.0)	11 (34.4)	0.9
Should be ligated both proximally and distally	32 (69.6)	127 (90.1)	25 (80.6)	27 (84.4)	0.039
Methods of thoracic duct ligation, if routinely ligated	16 (34.8)	34 (24.1)	9 (29.0)	8 (25.0)	0.5
Sutures	36 (78.3)	110 (78.0)	27 (87.1)	30 (93.8)	0.157
Metal clips	26 (56.5)	82 (58.2)	15 (48.4)	19 (59.4)	0.8
Plastic clips	20 (43.5)	88 (62.4)	16 (51.6)	11 (34.4)	0.011
Ligasure	4 (8.7)	6 (4.3)	5 (16.1)	4 (12.5)	0.085
**Postoperative - Diagnosis** *(Which features influence diagnosis of chyle leaks?)*					
>500mL of chest drain output within 24-hours in the absence of enteral feeding	34 (73.9)	66 (46.8)	16 (51.6)	19 (59.4)	0.013
Milky chest drain output	46 (100.0)	129 (91.5)	30 (96.8)	27 (84.4)	0.045
Clinically suspected leak					
Confirmed triglycerides ≥ 1.1 mmol/L or 19.8 mg/dl in chest drain fluid	40 (87.0)	118 (83.7)	29 (93.5)	32 (100.0)	0.056
Confirmed with presence of chylomicrons in chest drain fluid	38 (82.6)	127 (90.1)	28 (90.3)	28 (87.5)	0.6
Exclusion of anastomotic leak	39 (84.8)	80 (56.7)	21 (67.7)	15 (46.9)	0.002

**Postoperative - Severity** *(Which features are defining criteria severity of chyle leaks?)*					
Volume of chest drain output within 24-hours	45 (97.8)	130 (92.2)	28 (90.3)	31 (96.9)	0.397
Timing of chyle leak	22 (47.8)	33 (23.4)	11 (35.5)	11 (34.4)	0.016
Total duration of chyle leak	42 (91.3)	114 (80.9)	26 (83.9)	18 (56.2)	0.002
Chyle leak associated with low albumin and lymphopenia	39 (84.8)	90 (63.8)	20 (64.5)	19 (59.4)	0.043
Chyle leak associated with complications (i.e. pulmonary, anastomotic leak)	35 (76.1)	64 (45.4)	22 (71.0)	12 (37.5)	<0.001
**Postoperative - Management** *(What approaches you consider in management of chyle leaks?)*					
Step up approach (conservative > interventional > operative)	42 (91.3)	108 (76.6)	31 (100.0)	26 (81.2)	0.005
Early operative treatment for chyle leak is important	14 (30.4)	50 (35.5)	11 (35.5)	19 (59.4)	0.05
Imaging (i.e lymphangiogram, MRI) to locate chyle leak is preferable prior to re-operating	21 (45.7)	73 (51.8)	18 (58.1)	16 (50.0)	0.8
Non-operative management alone should be successful	24 (52.2)	57 (40.4)	16 (51.6)	13 (40.6)	0.414
Non-operative (i.e. conservative / interventional) methods should be attempted prior to operative treatment	37 (80.4)	109 (77.3)	29 (93.5)	25 (78.1)	0.044
Administration of octreotide as a part of non-operative management	24 (52.2)	34 (24.1)	14 (45.2)	12 (37.5)	0.002
Lymphangiogram and embolization of thoracic duct for treating chyle leak	30 (65.2)	102 (72.3)	20 (64.5)	23 (71.9)	0.7
Pleurodesis as a part of non-operative management	12 (26.1)	16 (11.3)	9 (29.0)	6 (18.8)	0.029

Supplementary Table 13 Summary of agreement of respondents from 3 of the modified Delphi process across the risk factors, intraoperative techniques, and postoperative diagnosis and management of chyle leaks domains stratified by surgeon volume

	**≤ 20, n=177**	**21 - 50, n=62**	**≥ 50, n=11**	**p-value**
**Risk factors** *(Do you consider these factors as risk factors for chyle leaks?)*				
Gender - Male	33 (18.6)	11 (17.7)	2 (18.2)	1.0
Tumor Location - Proximal / Middle	120 (67.8)	43 (69.4)	5 (45.5)	0.3
Body mass index, Low	56 (31.6)	21 (33.9)	4 (36.4)	0.9
Low pre-operative albumin	58 (32.8)	10 (16.1)	0 (0.0)	0.005
Use of neoadjuvant therapy	48 (27.1)	13 (21.0)	0 (0.0)	0.1
Use of neoadjuvant chemoradiotherapy*	113 (63.8)	37 (59.7)	6 (54.5)	0.7
Response from neoadjuvant therapy	72 (40.7)	22 (35.5)	3 (27.3)	0.6
Interval from neoadjuvant therapy to surgery	43 (24.3)	12 (19.4)	2 (18.2)	0.7
Abnormal spinal anatomy	124 (70.1)	50 (80.6)	11 (100.0)	0.035
Abnormal duct anatomy	162 (91.5)	61 (98.4)	11 (100.0)	0.1
Minimally invasive esophagectomy	17 (9.6)	6 (9.7)	0 (0.0)	0.6
Transhiatal esophagectomy	71 (40.1)	21 (33.9)	4 (36.4)	0.7
Lymphadenectomy	144 (81.4)	53 (85.5)	8 (72.7)	0.5
**Intraoperative** *(Do these intraoperative techniques minimize chyle leaks?)*				
Identifying thoracic duct during an esophagectomy is important				
Pre-operative lymphangiogram with indocyanine Green	133 (75.1)	47 (75.8)	8 (72.7)	1.0
Intra-operative fatty feed (i.e. cream, enteral feed)	40 (22.6)	9 (14.5)	5 (45.5)	0.1
Methylene blue or Indian ink added to the fatty feed	119 (67.2)	45 (72.6)	8 (72.7)	0.4
Intraoperative peritumoural injection with Indocyanine Green	32 (18.1)	10 (16.1)	2 (18.2)	0.5
Minimal access surgery	20 (11.3)	7 (11.3)	0 (0.0)	0.3
Thoracic duct should be ligated routinely	102 (57.6)	39 (62.9)	9 (81.8)	0.2
Should be ligated proximally below the level of azygos vein	139 (78.5)	53 (85.5)	9 (81.8)	0.5
Should be ligated distally in the lower thoracic cavity	58 (32.8)	19 (30.6)	5 (45.5)	0.6
Should be ligated both proximally and distally	145 (81.9)	56 (90.3)	10 (90.9)	0.6
Methods of thoracic duct ligation, if routinely ligated	43 (24.3)	20 (32.3)	4 (36.4)	0.4
Sutures	144 (81.4)	50 (80.6)	9 (81.8)	1.0
Metal clips	98 (55.4)	38 (61.3)	6 (54.5)	0.7
Plastic clips	90 (50.8)	35 (56.5)	10 (90.9)	0.0
Ligasure	15 (8.5)	4 (6.5)	0 (0.0)	0.5
**Postoperative - Diagnosis** *(Which features influence diagnosis of chyle leaks?)*				
>500mL of chest drain output within 24-hours in the absence of enteral feeding	101 (57.1)	29 (46.8)	5 (45.5)	0.3
Milky chest drain output	164 (92.7)	57 (91.9)	11 (100.0)	0.6
Clinically suspected leak				
Confirmed triglycerides ≥ 1.1 mmol/L or 19.8 mg/dl in chest drain fluid	155 (87.6)	54 (87.1)	10 (90.9)	0.9
Confirmed with presence of chylomicrons in chest drain fluid	156 (88.1)	55 (88.7)	10 (90.9)	1.0
Exclusion of anastomotic leak	114 (64.4)	35 (56.5)	6 (54.5)	0.5
**Postoperative - Severity** *(Which features are defining criteria severity of chyle leaks?)*				

Volume of chest drain output within 24-hours	165 (93.2)	58 (93.5)	11 (100.0)	0.7
Timing of chyle leak	56 (31.6)	20 (32.3)	1 (9.1)	0.3
Total duration of chyle leak	138 (78.0)	52 (83.9)	10 (90.9)	0.4
Chyle leak associated with low albumin and lymphopenia	120 (67.8)	41 (66.1)	7 (63.6)	0.9
Chyle leak associated with complications (i.e. pulmonary, anastomotic leak)	102 (57.6)	26 (41.9)	5 (45.5)	0.1
**Postoperative - Management** *(What approaches you consider in management of chyle leaks?)*				
Step up approach (conservative > interventional > operative)	144 (81.4)	53 (85.5)	10 (90.9)	0.6
Early operative treatment for chyle leak is important	71 (40.1)	19 (30.6)	4 (36.4)	0.4
Imaging (i.e lymphangiogram, MRI) to locate chyle leak is preferable prior to re-operating	88 (49.7)	34 (54.8)	6 (54.5)	0.8
Non-operative management alone should be successful	75 (42.4)	30 (48.4)	5 (45.5)	0.7
Non-operative (i.e. conservative / interventional) methods should be attempted prior to operative treatment	137 (77.4)	53 (85.5)	10 (90.9)	0.6
Administration of octreotide as a part of non-operative management	64 (36.2)	16 (25.8)	4 (36.4)	0.3
Lymphangiogram and embolization of thoracic duct for treating chyle leak	119 (67.2)	47 (75.8)	9 (81.8)	0.3
Pleurodesis as a part of non-operative management	36 (20.3)	6 (9.7)	1 (9.1)	0.1

Supplementary Figure 1 PRISMA diagram of included studies into the scoping review

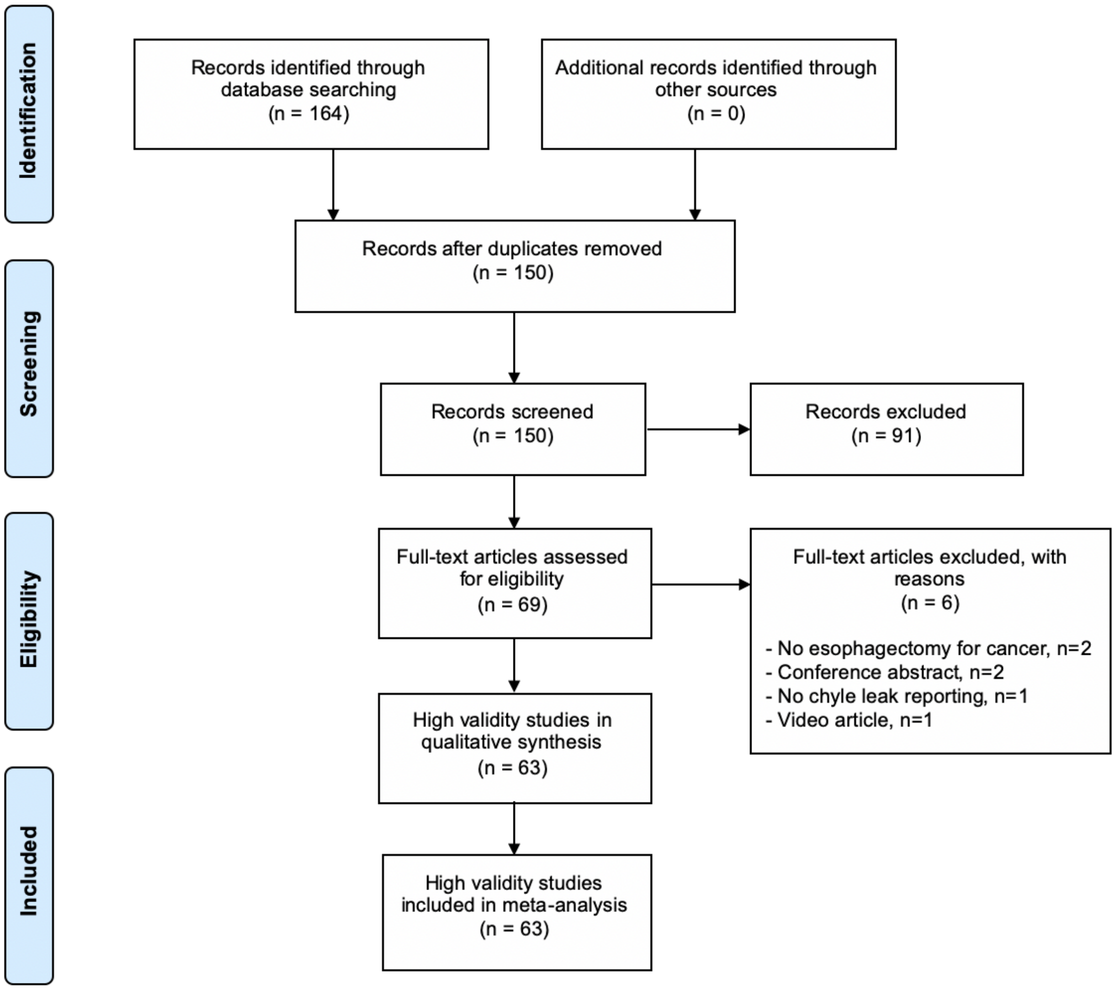

Supplementary Figure 2 Pooled chyle leak rates across included studies from the scoping review

Study	Events	Total	Proportion	95%−CI	Weight (fixed)	Weight (random)
Orringer 1988	11	320	0.03	[0.02; 0.06]	1.2%	2.9%
Bolger 1991	11	537	0.02	[0.01; 0.04]	1.2%	2.9%
Dougenis 1992	10	255	0.04	[0.02; 0.07]	1.1%	2.9%
Alexiou 1998	21	523	0.04	[0.03; 0.06]	2.3%	3.1%
Dugue 1998	23	850	0.03	[0.02; 0.04]	2.5%	3.1%
Merigliano 2000	19	1787	0.01	[0.01; 0.02]	2.1%	3.1%
Bonavina 2001	3	316	0.01	[0.00; 0.03]	0.3%	2.2%
Lagarde 2005	20	536	0.04	[0.02; 0.06]	2.2%	3.1%
Hayden 2007	6	129	0.05	[0.02; 0.10]	0.6%	2.6%
Schumacher 2007	10	409	0.02	[0.01; 0.04]	1.1%	2.9%
Benedix 2010	3	56	0.05	[0.01; 0.15]	0.3%	2.2%
Lai 2011	8	653	0.01	[0.01; 0.02]	0.9%	2.8%
Guo 2012	8	135	0.06	[0.03; 0.11]	0.8%	2.8%
Shah 2012	34	892	0.04	[0.03; 0.05]	3.7%	3.2%
Li 2013	306	10574	0.03	[0.03; 0.03]	33.3%	3.3%
Mishra 2013	9	104	0.09	[0.04; 0.16]	0.9%	2.8%
Fujita 2014	20	521	0.04	[0.02; 0.06]	2.2%	3.1%
Hou 2014	17	1804	0.01	[0.01; 0.02]	1.9%	3.1%
Kim 2014	57	1514	0.04	[0.03; 0.05]	6.1%	3.3%
Gupta 2015	4	45	0.09	[0.02; 0.21]	0.4%	2.3%
Miao 2015	34	1290	0.03	[0.02; 0.04]	3.7%	3.2%
Brinkmann 2016	17	906	0.02	[0.01; 0.03]	1.9%	3.1%
Lin 2017	5	296	0.02	[0.01; 0.04]	0.6%	2.5%
Weijs 2017	78	371	0.21	[0.17; 0.26]	6.9%	3.3%
Oguma 2018	11	290	0.04	[0.02; 0.07]	1.2%	2.9%
Ohkura 2018	19	371	0.05	[0.03; 0.08]	2.0%	3.1%
Ohkura 2018	24	294	0.08	[0.05; 0.12]	2.5%	3.1%
Batool 2019	9	193	0.05	[0.02; 0.09]	1.0%	2.8%
Schurink 2019	44	198	0.22	[0.17; 0.29]	3.8%	3.2%
Malibary 2020	7	70	0.10	[0.04; 0.20]	0.7%	2.7%
Farran 2021	13	274	0.05	[0.03; 0.08]	1.4%	3.0%
Higuchi 2021	12	390	0.03	[0.02; 0.05]	1.3%	3.0%
Jeon 2021	26	605	0.04	[0.03; 0.06]	2.8%	3.2%
Milito 2021	50	992	0.05	[0.04; 0.07]	5.3%	3.2%
**Fixed effect model**		**28500**	**0.04**	**[0.04; 0.04]**	**100.0%**	**--**
**Random effects model**			**0.04**	**[0.03; 0.05]**	**--**	**100.0%**

Heterogeneity: $I^2 = 94\%$, $\tau^2 = 0.6384$, $p < 0.01$

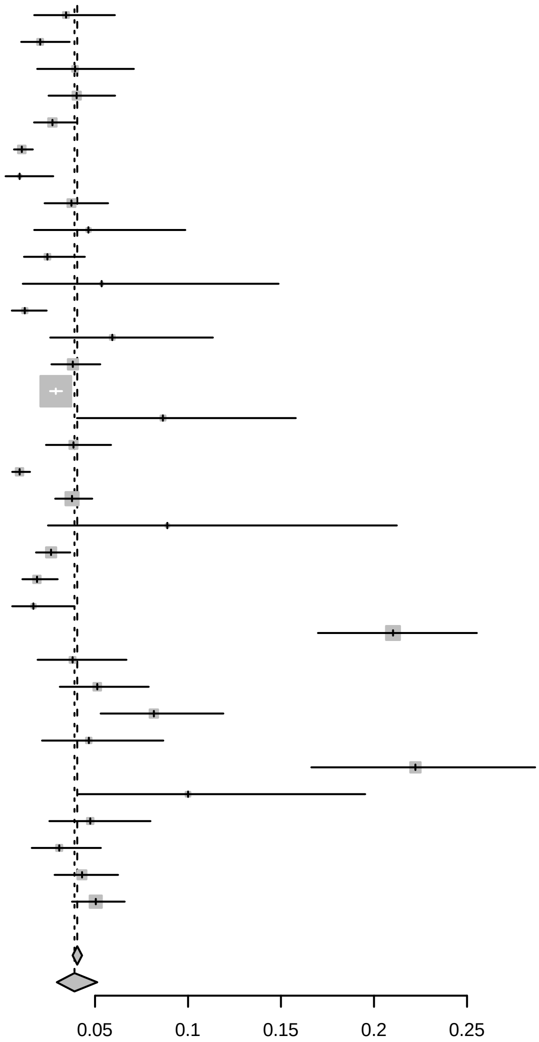

Supplement: Supplementary file 1 [file as9-3-e192-s001.pdf]
